# Long-term oral intake of *Panax ginseng* improves hypomagnesemia, hyperlactatemia, base deficit, and metabolic acidosis in an alloxan-induced rabbit model

**DOI:** 10.22038/ijbms.2019.33223.7936

**Published:** 2019-06

**Authors:** Gareeballah Osman Adam, Gi-Beum Kim, Sei-Jin Lee, Hee-Ryung Lee, Shang-Jin Kim, Hyung-Sub Kang, Jin-Shang Kim

**Affiliations:** 1 Department of Veterinary Pharmacology and Toxicology, College of Veterinary Medicine, Chonbuk National University, Iksan Campus, 79 Gobong-ro, Iksan-si, Jeollabuk-do 54596 Republic of Korea; 2 Department of Veterinary Medicine and Surgery, College of Veterinary Medicine, Sudan University of Science and Technology, Hilat Kuku, Khartoum, Sudan; 3 Korea Basic Science Institute Jeonju Centre, Deokjin gu, Jeonju-si, Jeollabuk do 54896, Republic of Korea

**Keywords:** Acidosis, Glycated hemoglobin A1c, Hyperlactatemia, Hypomagnesemia, Panax ginseng, Type 1 diabetes

## Abstract

**Objective(s):**

*Panax ginseng* (PG) widely used for its various pharmacological activities, including effects on diabetes and its complications. This study aims to investigate the effect of PG on mortality-related hypomagnesemia, hyperlactatemia, metabolic acidosis, and other diabetes-induced abnormalities.

**Materials and Methods:**

Type 1 diabetes was induced by IV injection of alloxan monohydrate 110 mg/kg into New Zealand white rabbits weighing 2-2.5 kg. PG was supplied in drinking water for 20 weeks. The effects of the PG treatment on diabetes were evaluated through hematological and biochemical analysis including ELISA assays for insulin and glycated haemoglobin A1c (HBA1c) before and after PG extract was supplied.

**Results:**

The serum glucose, insulin, and HBA1c levels were significantly improved after the PG treatment compared to those found before PG treatment. In addition, Mg^2+^, lactate, and base deficit, and acidosis was significantly enhanced in treated rabbits. Moreover, PG showed hepato- and renoprotective effect. Likewise, electrolytes, lipid and protein profile were improved.

**Conclusion:**

The biochemical and hematological analysis data demonstrate that the PG is effective to alleviate the diabetes serious signs.

## Introduction

Globally, diabetes becoming a serious disease associated with health and life-style factors, such as obesity, stress, overeating, and lack of exercise (1). The main early symptom of diabetes is a high blood glucose level (hyperglycemia). Hyperglycemia can lead to a wide range of metabolic disorders of carbohydrates, proteins, fats, and electrolytes (2, 3). 

The treatment of diabetes depends on insulin injections, diet, and exercise. However, Insulin injection, the major therapy for type 1 diabetes, can produce several side effects. Therefore, numerous studies had been performed in order to find active herbal ingredients (4, 5).

*Panax ginseng* (PG), as an alternative medicine, has been widely used in East Asian countries. PG and its ginsenosides possess multiple pharmacological actions for treating various diseases and inflammatory conditions including liver regeneration, cerebral ischemia (6, 7).

The active ingredients in red ginseng are classified into saponin components (ginsenosides) and non-saponin components (the polyacetylene compounds, panaxatriol and panaxadiol, acidic polysaccharides, and amino acids), these ingredients are responsible for immune stimulation, and prevention of metabolic diseases such as diabetes (8, 9).

In a comparative study between the effects of ginseng roots and berry on glycemic index, Dey and his co-workers reported that ginseng root has potential anti-hyperglycemic activity (10). A recent study suggested that PG improves high level of glucose by a plethora of mechanisms including regulation of insulin sensitivity, modulating gastrointestinal absorption, and anti-inflammatory effect (11).

In the present study, therapeutic actions of red ginseng were evaluated in an alloxan-induced type 1 diabetic rabbit model using some hematological and biochemical parameters. In addition, this study focuses on the prevention of hypomagnesemia, hyperlactatemia, and base excess deficit when PG supplied in drinking water for a long duration. 

## Materials and Methods

***Experimental animals***

A total of 10 male New Zealand white rabbits weighing 2.5- 3.0 kg were used in the study. All rabbits were separately kept in cages with wide square mesh at the bottom to avoid coprophagy and maintained under controlled conditions of humidity, temperature (22±2 ^°^C) and 12-hr light and dark cycle. Food and water were provided *ad libitum*. The rabbits were fasted for 12 hr prior to the experiment, but allowing free access to water only. The experimental protocols were approved by the Institutional Animal Ethics Committee. All experimental protocols (CBU2013-0010) were approved by the Committee on the Care of Laboratory Animal Resources, Chonbuk National University and were conducted in accordance with the Guide for the Care and Use of Laboratory.

***Induction of experimental diabetes - Procedure for injecting alloxan monohydrate***

Rabbits were made diabetic by IV injection of 110 mg/kg body weight of alloxan monohydrate (A7413, Aldrich) (10). Before giving alloxan, the normal blood glucose levels of all rabbits were estimated using ACCU-CHEK® Active, Roche Diagnostics, Mannheim, Germany. After 2 hr of alloxan injection, 5% of Dextrose injected to the all diabetic rabbits IP to prevent a hypoglycemic condition. After 72 hr of alloxan injection, the serum glucose levels of all surviving rabbits were determined by Model 7020 autoanalyzer (Hitachi, Tokyo, Japan).

***PG treatment***

 PG alcohol extract was obtained from Jinan institute (voucher no. 75720). In this study, the red ginseng extract was prepared into 9 l 80% (v/v) alcohol at 80 ^°^C for 8 hr by 1st extraction, and this extraction was done into 6 l 80% (v/v) alcohol at 80 ^°^C for 8 h by 2^nd^ extraction. The treated group was given as 0.66 mg/ml of PG in drinking water; the red ginseng in drinking water was changed every day over 20 weeks and was prepared just before the water change. Water was available *ad libitum*. During the study duration, the health conditions of rabbits were monitored every day, and the body weights were measured every week. No signs of toxicity were observed.

***Biochemical analysis***

 Blood was collected from the ear marginal vein. A Nova Stat Profile^®^ pHOx^®^ Ultraanalyzer (NOVA Biomedical Corp, Waltham, MA, USA) was used to measure blood gas, electrolytes, and anion gap. After clotting, blood serum was separated by centrifugation (Daihan, Seoul, South Korea) at 3000 rpm for 20 min. The levels of glucose (Glu), enzymes, lipids, and proteins were analyzed using a Model 7020 autoanalyzer (Hitachi, Tokyo, Japan). 

***Serum insulin and glycated haemoglobin A1c (HbAlc) Measurements***

Fasting serum insulin levels were measured with a rat insulin enzyme-linked immune absorbent assay kit (LifeSpan BioSciences, Inc., LS-F21890) according to the manufacturer’s protocol. HbA1c ELISA kit (ALPCO Diagnostics, Windham, NH, USA) were quantitated using commercially available kits according to the manufacturers’ protocols.

***Statistical analysis***

 Data are expressed as mean±standard errors of the mean (SEMs). Differences between pre (normal) and post (diabetic) rabbits were evaluated by one-way paired *t*-test whereas between post diabetic and treated rabbit’s one-way analysis of variance (ANOVA) with the Bonferroni *post hoc* test or by calculation of Spearman’s rank correlation coefficient, as appropriate, using Prism 5.03 (GraphPad Software Inc., San Diego, CA, USA). Statistical significance was set at *P<*0.05.

## Results

***Serum glucose, insulin, and glycated HBA1C concentration***

**Figure 1:**
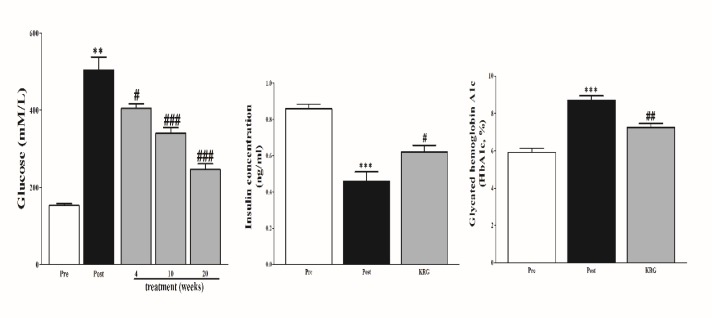
Effect of *Panax ginseng* on serum glucose, insulin, and glycated haemoglobin A1c. The data are reported as means±SEMs (n=10). *: *P*<0.05; **: *P*<0.01; and ***: *P*<0.001, paired student’s *t*-test pre (normal rabbit) vs post (diabetic rabbits); #: *P*<0.05; ##: *P*<0.01; and ###: *P*<0.001, Bonferroni *post hoc* test following one-way ANOVA post (diabetic) vs 4, 10, and 20 weeks of treatment for glucose, and #: *P*<0.05; ##: *P*<0.01; and ###: *P*<0.001 paired student’s* t*-test post (diabetic) 20 weeks after treatment for insulin and glycated haemoglobin A1c

To test the effects of PG on diabetes, serum glucose, insulin, and glycated HBA1C were measured (Figure 1). Diabetic rabbits showed a significant increase in the levels of glucose compared to normal rabbits. However, after we supplied PG in water, the glucose levels were substantially decreased in a time-dependent manner on 4^th^, 10^th^, and 20^th^ week, respectively compared to diabetic rabbits. In addition, the insulin and HBA1c were significantly decreased and increased, respectively in diabetic rabbits compared to normal rabbits. Regardless, PG-treated rabbits showed a significant improvement after 20 weeks of treatment. 

**Figure 2:**
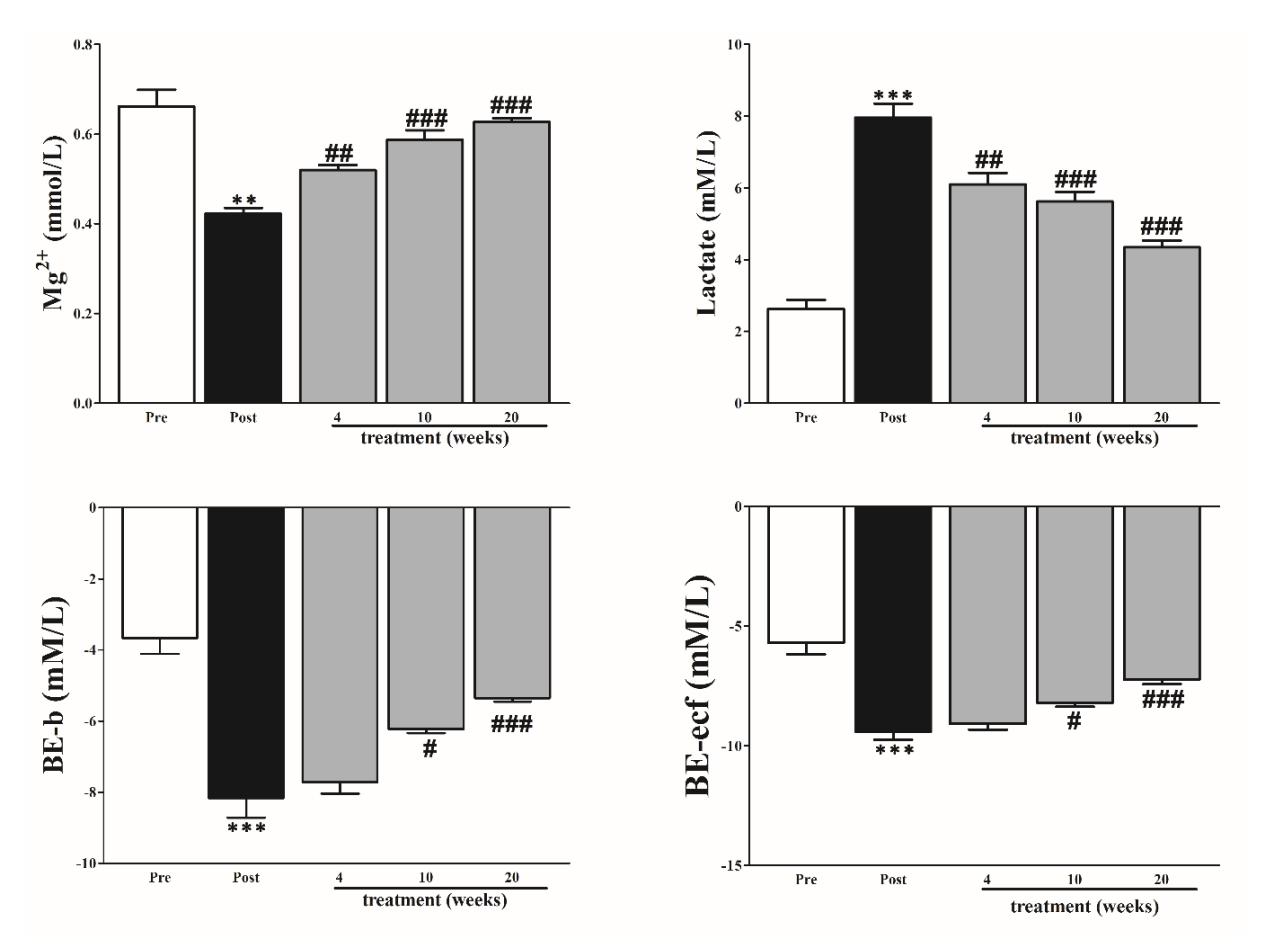
Effect of *Panax ginseng* on blood magnesium (Mg^2+^), lactate, base excess-blood (BE-b), and base excess extra-cellular fluid (BE-ecf). The data are reported as means±SEMs (n=10). *: *P*<0.05; **: *P*<0.01; and ***: *P*<0.001, paired student’s *t*-test pre (normal rabbit) vs post (diabetic rabbits); #: *P*<0.05; ##: *P*<0.01; and ###: *P*<0.001, Bonferroni *post hoc *test following one-way ANOVA post (diabetic) vs 4, 10, and 20 weeks of treatment

**Figure 3:**
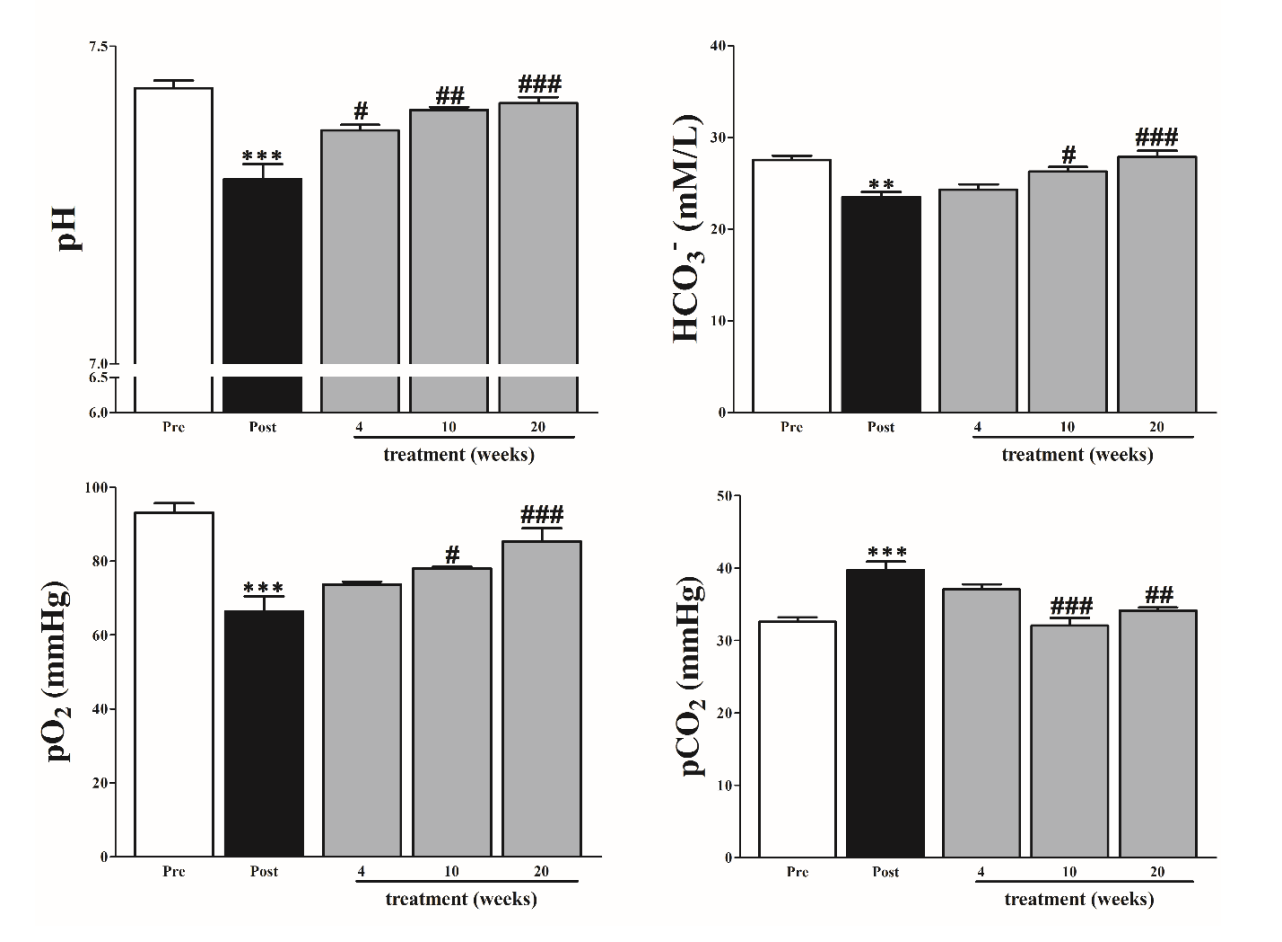
Effect of *Panax ginseng* on pH, bicarbonate (HCO_3-_), partial pressure of oxygen (pO_2_), partial pressure of Carbon dioxide (pCO_2_). The data are reported as means±SEMs (n=10). *: *P*<0.05; **: *P*<0.01; and ***: *P*<0.001, paired student’s *t*-test pre (normal rabbit) vs post (diabetic rabbits); #: *P* < 0.05; ##:* P*<0.01; and ###: *P*<0.001, Bonferroni *post hoc* test following one-way ANOVA post (diabetic) vs 4, 10, and 20 weeks of treatment

**Table 1:** Effect of *P. ginseng* on lipid and protein profile

			Treatment (weeks)		
	Pre	Post	4	10	20
HDL (mg/dL)	24.7±0.4	14.5±0.9***	16.5±0.9	23.3±1.2###	24.0±0.8###
T-CHO (mg/dL)	61.7±0.0	211.5±4.9***	168.8±5.2###	92.5±4.8###	78.7±0.7###
TG (mg/dL)	127.3±0.5	223.8±19.3**	169.2±6.5#	143.2±3.9###	156.0±4.8##
LDL (mg/dL)	37.4±2.1	142.3±5.3***	117.2±4.7##	81.33±5.8###	49.0±2.4###
T-PRO (mg/dL)	6.7±0.1	4.6±0.4***	6.0±0.3#	6.3±0.4##	6.6±0.5#

High density lipoprotein (HDL), total cholesterol (T-CHO), low density lipoprotein (LDL), triglyceride (TG), and total protein (T-PRO). The Data are reported as means±SEMs (n = 10).

* *: P *<* 0.05; *

** *: p *< 0.01*; *and

*** *: P *< 0.001, paired student’s t-test pre (normal rabbit) vs post (diabetic rabbits);

# *: P *< 0.05*; *

## *: P *< 0.01*; *and

### *: P *< 0.001, Bonferroni post hoc test following one-way ANOVA post (diabetic) vs 4, 10, and 20 weeks of KRG treatment.

**Table 2:** Effect of *P. ginseng* on liver and kidney function

			Treatment (weeks)		
	Pre	Post	4	10	20
AST	17.2±3.2	108.3±5.8**	81.2±2.6#	53.0±8.2###	38.2±3.0###
ALT	29.4±2.0	104.0±0.6***	74.3±1.7###	68.5±0.6###	41.3±2.2###
ALP	174.3±7.8	328.0±8.2***	325.0±11.6	302.3±5.1###	194.0±4.7###
LDH	105.5±4.4	350.0±9.7***	273.8±20.8##	117.3±2.6###	140.3±4.5###
CRE	0.67±0.04	1.20±0.05**	0.95±0.05	0.93±0.09	0.60±0.06###
BUN	12.7±0.8	39.7±0.9***	32.7±1.9#	26.9±2.4###	19.8±0.4###
UA	0.15±0.02	0.27±0.04	0.22±0.03	0.20±0.04	0.20±0.04
CK	474.0±32.3	836.0±40.3**	483.8±32.5###	418.3±30.3###	348.5±17###

Aspartate aminotransferase (AST), alanine aminotransaminase (ALT), (LDH) alkaline phosphatase (ALP) lactate dehydrogenase, creatinine (CRE), blood urea nitrogen (BUN), uric acid (UA), and creatine kinase (CK). The data are reported as means±SEMs (n = 10).

* *: P *< 0.05*; *

** *: P *< 0.01*; *and

** * *: P *< 0.001, paired student’s t-test pre (normal rabbit) vs post (diabetic rabbits);

# *: P *< 0.05*; *

## *: P *< 0.01*; *and

### *: P *< 0.001, Bonferroni post hoc test following one-way ANOVA post (diabetic) vs 4, 10, and 20 weeks of KRG treatment.

**Table 3:** Effect of *P. ginseng* on electrolytes and Haemoglobin

			Treatment (weeks)		
	Pre	Post	4	10	20
T-Mg^2+^	2.27±0.03	1.77±0.04***	2.35±0.06###	2.15±0.06##	2.20±0.05###
Ca^2+^	1.5±0.03	0.9±0.06	1.6±0.04	1.5±0.02	1.5±0.01
Na^+^	140.0±0.6	121.2±2.1^**^	136.5±2.0^###^	134.3±1.3^###^	140.7±0.6^###^
K^+^	5.5±0.4	4.0±0.2^*^	4.2±0.2	5.2±1.7##	5.8±0.2^###^
Cl^-^	106.0±0.2	92.9±0.4^***^	98.7±1.2	102.4±1.6##	100.9±2.4^#^
Hb	12.4±0.2	9.5±0.1^***^	13.1±0.2^###^	12.1±0.1###	13.7±0.7^####^

Total Magnesium (T-Mg^2+^) , Calcium (Ca^2+^) Sodium (Na^+^), Potassium (K^+^), Chloride (Cl^-^), and Hemoglobin (Hb) in blood. The data are reported as means±SEMs (n = 10).

* *: P *< 0.05*; *

** *: P *< 0.01*; *and

*** *: P *< 0.001, paired student’s t-test pre (normal rabbit) vs post (diabetic rabbits);

# *: P *< 0.05*;*

## *: P *< 0.01*; *and

### *: P *< 0.001, Bonferroni post hoc test following one-way ANOVA post (diabetic) vs 4, 10, and 20 weeks of KRG treatment.

***Blood Mg*** ^2+^ ***, lactate, and base excess***

Effects of PG on Magnesium (Mg^2+^), lactate, base excess-blood (BE-b), and base excess extra-cellular fluid (BE-ecf) were investigated in rabbits before and after PG supply (Figure 2). Variable differences between rabbits were observed, Mg^2+ ^and lactate levels were significantly reduced and elevated, respectively. As expected after PG was applied, magnesium and lactate levels were considerably improved over the course of the treatment. Besides, base excess showed a hazardous decrease in diabetic rabbits compared to normal rabbits. Importantly, the base deficit was positively improved after PG was provided. 

***pH and blood gases***

The levels of pH and blood gases showed a significant variation before and after treatment. Comparing to normal rabbits, diabetic rabbits exhibited significantly decreased levels of pH, bicarbonate (HCO_3_), and partial pressure of oxygen (*p*O_2_), whereas partial pressure of Carbon dioxide (*p*CO_2_) was significantly increased. The result draws a typical picture of metabolic acidosis. Interestingly, PG intake enhanced the abnormalities of pH and gases over the treatment period (Figure 3). 

***Lipid and protein profile***

Total cholesterol (T-CHO), low-density lipoprotein (LDL), triglyceride (TG), and total protein (T-PRO) levels in the serum were significantly increased in the diabetic rabbit vs normal rabbits. On the other hand, high-density lipoprotein (HDL) was significantly decreased in diabetic rabbits compared to normal rabbits. However, after PG treatment, dyslipidemia, HDL level, and T-PRO level were significantly showed better results compared to before treatment with PG as listed in Table 1.

***Liver and kidney function***

To examine the efficacy of the PG treatment on diabetes in a diabetic rabbit model liver function tests such as serum aspartate aminotransferase (AST), alanine aminotransaminase (ALT), lactate dehydrogenase (LDH) and alkaline phosphatase (ALP) were analyzed as shown in Table 2. In addition, serum creatinine (CRE), blood urea nitrogen (BUN), uric acid (UA), and creatine kinase (CK) to examine the renal function before and after treatment (Table 2). In diabetic rabbits, the levels of these enzymes and indicators were significantly increased compared to normal rabbits. Despite that, treated rabbits ensured the pharmacological effect of PG by improving liver and kidney function through enhancement of the levels of these parameters. Although UA shows a reduction in diabetic rabbits and improvement after PG was supplied, however, these changes were insignificant. 

***Electrolytes and haemoglobin***

It was shown that the levels of total magnesium (T-Mg^2+^) in serum, Calcium (Ca^2+^) sodium (Na^+^), potassium (K^+^), chloride (Cl^-^), and hemoglobin (Hb) in blood were significantly reduced in diabetic rabbits compared to normal rabbits. However, in rabbits subjected to PG treatments, the electrolytes and Hb concentrations showed significant enhancement as presented in Table 3. 

## Discussion

The data demonstrated that PG showed significant effects in diabetic rabbits when supplied to rabbits in drinking water over 20 weeks. The levels of Insulin, HBA1c, glucose levels were substantially improved. Liver and kidney functions, and lipoprotein profile were significantly improved. Importantly, PG improved hypomagnesemia metabolic acidosis, base deficit, hyperlactatemia, and acid-base balance. 

Diabetic rabbits showed a significant increase in glucose level which is due to destruction in β- cells of pancreas producing hyperglycemia (12). However, after PG treatment, the glucose level was significantly reduced starting from the 10^th^ week of treatment. PG is proved to contain ingredients that maintain sugar glucose and enhancing insulin resistance (8). 

Decreased level of insulin and an increased level of HbA1c in diabetic rabbit serum were observed in this study. Insulin deficiency due to pancreas destruction is the main feature of diabetes, HbA1c is a marker for diagnosis of diabetes which is more important than glucose as HbA1c remains high regardless glucose level in diabetic patients (13, 14). However, in PG-treated rabbits, the insulin level was significantly increased compared to those in diabetic rabbits as well as HbA1c level was significantly reduced after treatment with PG. Our result compatible with numerous studies (15-17) reported that the hypoglycemic effect of red ginseng could be attributed to the ability of red ginseng to promote the insulin secretion, improve glycemic index, and enhance α-amylase activity. Although some markers and indicators mentioned herein this discussion were not measured in this experiment, we think that if they were measured, would have a positive response in the PG treated group.

Hyperglycemia is well associated with hypomagnesemia which had been reported to cause oxidative stress in chronic conditions such as diabetes (18, 19). Base excess has been used as an indicator of metabolic acidosis in metabolic conditions, blood lactate as an indicator of energy negative balance and oxidative stress, these have been reported to show high levels in insulin-resistant patients compared to normal people (20, 21). All these disturbances were typically observed in our study in the diabetic rabbit which can be attributed to insulin resistance complications in different tissues and fluids, and osmotic renal loss. Improvement of insulin level and reduced glucose level due to PG might help in reversing this result after a long course of intake in diabetic rabbits, this supported by several reports, Jung *et al*. observed that ginseng prevents against nephrotoxicity which is associated with a change in renal osmolarity (22). We think this effect promote kidney function and then reduce Mg^2+^ loss, hence prevent hypomagnesemia in diabetic rabbit. Low base excess as an indication of tissue hypoxia was used as a prognostic criterion for severity of some diseases (23). We discussed several evidences proved that PG improved lung inflammation and it’s complication such as respiratory acidosis and blood oxygen perfusion. Furthermore, the ability of PG to clear blood lactate in treated group is considered a sign of it’s potential to improve oxygen to tissues because the higher lactate level is associated with hypoxia (24). Although lactate is an important indicator of morbidity and mortality in emergency unit, however, BE was proven by a recent study to be more important than lactate in the evaluation of disease severity (25). 

The result indicates that the levels of blood CO_2_ and *p*CO_2_ were decreased in diabetic rabbits before PG treatment. In addition, the levels of pH, O_2_, HCO_3_^-^, pO_2_, and SO_2_ were decreased in diabetic rabbits before PG treatment as well. These abnormalities indicate metabolic and respiratory acidosis as a consequence of diabetes, HCO_3_^-^ level lower than 18 mmol per l, pH less than 7.3, anion gap more than 16 mmol per l, and glucose level more than 250 mg/dl are collectively used for diagnosing diabetic ketoacidosis (26, 27). However, long course administration of PG revealed a significant improvement in acid-base balance and related acidosis in rabbits. Our result consisted with previous studies stated that fermented red ginseng prevents metabolic disturbance and dyslipidemia via improving insulin receptor substrate and glucose transport type 4 in rats, red ginseng has been also proved to promote an insulin-like acid peptide in diabetic condition (28, 29). Red ginseng was also reported to mitigate the inflammation in the lung via AMPK inhibition (30). 

Impairment of proteins and lipids metabolism had been associated with diabetes (31). In this study, the diabetic rabbits showed elevated levels of TG, TC, and LDL, but decreased level of HDL. Hypertriglyceridemia, and hypercholesterolemia are major risk factors for diabetes with respect to the development of atherosclerosis and coronary heart disease (32). The long duration intake of PG lowered the levels of LDL, TG, and T-CHO and enhanced the levels of T-PRO and HDL, this result can be attributed to the antioxidant potential and NO production which modulate blood circulation. Furthermore, saponin content of ginseng is known to reduce the atherogenesis in rabbits via PPARα which activate β oxidation of fatty acids, hence reduces lipid accumulation (28, 33, 34). 

Our result showed that the levels of liver enzymes were significantly elevated after diabetes induction. The elevated plasma levels of ALT, AST, ALP, and LDH indicate liver stress in diabetic animals which has been well documented by Go *et al *(32). Interestingly, PG successfully reduced the levels of liver indicators showing hepatoprotective effect. This inconsistent with other observation reported by Kim *et al.* (35) stated that AST and ALT significantly decreased in diabetic rats upon oral administration of red ginseng which might be due to antioxidantion properties. The effect of PG on liver function and dyslipidemia through its antioxidant potential and reduction of fatty acid synthase thought to be behind this positive effect (33, 35-37).

The result also exhibited that the serum levels of CRE, BUN, and UA were significantly increased in diabetic rabbits. A recent study showed that one of the consequences of diabetes are acute renal failure, extracellular dehydration, and protein catabolism (38). On contrast, these indicators were improved when PG supplied, these findings coincided with a recent study reported that ginseng treatment in severe nephropathy improves renal function via decreasing autopathic vacuoles and genes such as Beclin-1 (39). As a progress of diabetes, a state of hyperproteinemia was evidenced, same observation was shown in our result which may resulted from either hyper filtration induced diabetic nephropathy and/or increased protein catabolism (40). 

In addition, data showed that the levels of Hb and CRE were significantly decreased in diabetic rabbits. Hb as an indicator of anemia and low creatinine as an indicator of kidney diseases are associated with diabetes complication (41, 42). PG used in this study improved the function of kidney and blood profile, several studies we discussed supported the positive effect of red ginseng on kidney and blood circulation (43).

The result indicated that electrolytes levels were variably disturbed. This observed turmoil in electrolytes could have been produced by dehydration or as a result of osmotic diuresis due to hyperglycemia and kidney dysfunction caused by diabetes, the effect of diabetes on kidney was discussed above in this study. In addition, the ability of PG to improve renal function was well-documented (43).

## Conclusion

*P. ginseng* plays an important role in lowering glucose level, promoting insulin secretion, alleviating diabetes complications such as hypomagnesemia, hyperlactatemia, acid-base disturbance, and dyslipidemia. However, this study did not show effect of long course treatment with PG on histopathology of organs. 

## Conflicts of Interest

There are no conflicts of interest
